# *Porongurup*, a new genus of pselaphine staphylinid beetles from Western Australia (Coleoptera, Staphylinidae, Pselaphinae, Faronitae)

**DOI:** 10.3897/zookeys.881.39535

**Published:** 2019-10-17

**Authors:** Su-Ho Choi, Donald S. Chandler, Jong-Seok Park

**Affiliations:** 1 Chungbuk National University, 1 Chungdae-ro, Seowon-gu, Cheongju-si, Chungbuk-do 28644, South Korea Chungbuk National University Cheongju South Korea; 2 Department of Biological Sciences, University of New Hampshire, Durham, NH, 03824, USA University of New Hampshire Durham United States of America

**Keywords:** biodiversity, biogeography, Faronini, taxonomy

## Abstract

A new genus and three new species of pselaphine staphylinid beetles, supertribe Faronitae, from Western Australia are described as follows: *Porongurup***gen. nov.** is based on *Porongurup angulatus***sp. nov.**, with the two additional new species, *Porongurup clarkei***sp. nov.** and *Porongurup tenuis***sp. nov.** Illustrations of their habitus, and major diagnostic characters as well as a distribution map are included. A key to the species is provided.

## Introduction

The supertribe Faronitae consists of 29 genera worldwide, with 13 genera being found in Australia and New Zealand (Park and Chandler 2017). At this time only three faronite genera [*Sagola* Sharp, 1874 (9 spp.), *Logasa* Chandler, 2001 (3 spp.), and *Nornalup* Park & Chandler, 2017 (3 spp.)] are known from Australia, although eastern Australia does have a complex and largely unknown fauna of Faronitae that is currently under study by J.-S. Park and colleagues. This is the second paper treating the separate and unique fauna of Faronitae from the southwestern corner of Australia, following the treatment of *Nornalup* by Park and Chandler (2017).

In the collections of Faronitae being examined as part of a treatment of the fauna of Australia, 31 specimens were segregated into three species that shared a combination of discrete diagnostic characters, with that combination separating them from all other known faronite genera and supporting the creation of a new genus. This new genus can be recognized by the following combination of characters: rostrum with linear frontal sulcus, abdominal segment VI approximately twice as long as V, and the male genitalia have the median lobe of the male genitalia elongate and narrow, culminating in an acute apex. The included species of this genus are known only from the southwestern corner of Western Australia.

The two faronite genera known from extreme southwestern Australia, *Porongurup* gen. nov. and *Nornalup*, are apparently restricted to this area, which is known as a global biodiversity hotspot that has a climatically isolated flora and fauna (Slater 1975, Coates and Atkins 2001, Hopper and Gioia 2004, Slatyer et al. 2007, Park and Chandler 2017). Members of *Porongurup* gen. nov. and *Nornalup* have been collected together in samples from this area, and apparently share the same microhabitats. However, the species of *Porongurup* have never been recorded together in samples, indicating differences in habitat preferences for these species.

## Materials and methods

Thirty-one specimens were examined from the following collections: Field Museum of Natural History (**FMNH**), Chicago, Illinois, USA, and the University of New Hampshire Insect Collection (**UNHC**), Durham, New Hampshire, USA. Six specimens were mounted on permanent slides to aide in observation of the internal characters and the fine external characters that are not apparent when using a dissecting microscope. Permanent microscopic slides were prepared using the techniques described by Hanley and Ashe (2003). Terminology for the foveal system and nomenclature follows Chandler (2001). Decimal Degrees were used for the format of geographical coordinates. Holotypes are deposited in the Western Australian Museum (**WAM**), Perth, Western Australia, Australia, and paratypes are deposited in the Field Museum of Natural History, the Western Australian Museum, the Australian National Insect Collection (**ANIC**), Canberra, ACT, Australia, the University of New Hampshire Insect Collection, Durham, New Hampshire, USA, and the Chungbuk National University Insect Collection (**CBNUIC**), Cheongju, Chungbuk-do, South Korea (indicated parenthetically). Specimen label data for the holotypes is transcribed verbatim. Data from the paratypes are standardized for consistency. The map of Australia is based on an image from SimpleMappr (Shorthouse 2010), that was subsequently modified to indicate localities of the specimens.

## Systematics

### Porongurup gen. nov.

Taxon classificationAnimaliaColeopteraStaphylinidae

AA67389E-95BF-5C97-AC1E-C31AF8A905BE

http://zoobank.org/362C6CA0-A334-4897-927B-B40A527B0118

#### Type species.

*Porongurup angulatus* sp. nov., herein designated.

#### Diagnosis.

**Figure 1.**
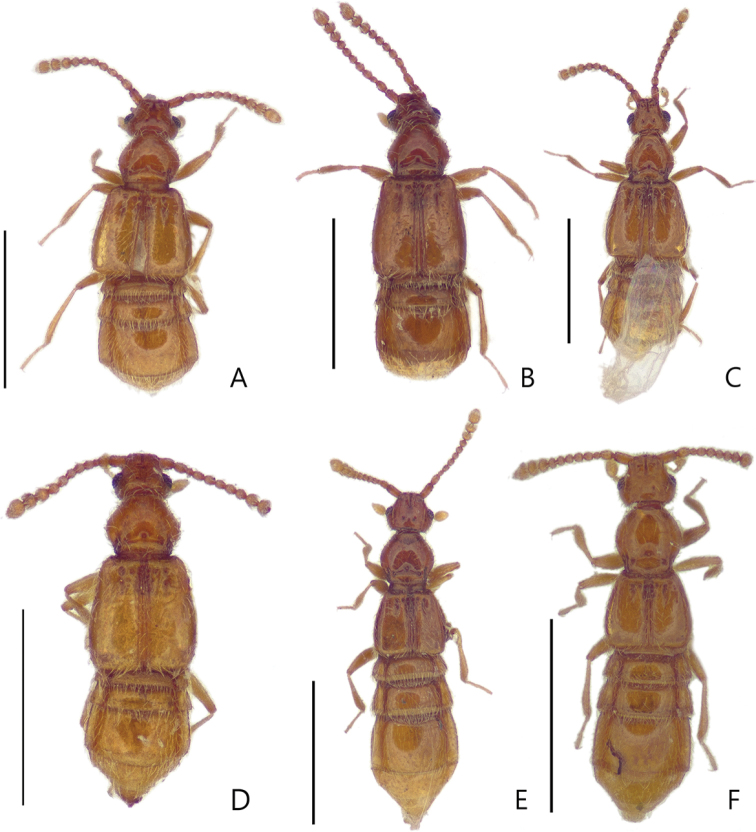
Male habitus photos, dorsal view **A***Porongurup angulatus* sp. nov. **B***P. clarkei* sp. nov. **C***P. tenuis* sp. nov. Female habitus, dorsal view **D***P. angulatus* sp. nov. **E***P. clarkei* sp. nov. **F***P. tenuis* sp. nov. Scale bar: 1 mm.

Members of this genus are easily separated from other faronite genera by the following combination of characters: rostrum with linear frontal sulcus (Fig. 3A); pronotum with median antebasal foveae, outer basolateral foveae, and inner basolateral foveae; prosternum with lateral mesocoxal foveae, and median procoxal foveae (Fig. 3B); mesoventrite with lateral mesosternal foveae and promesocoxal foveae (Fig. 3C); metaventrite with lateral metasternal foveae and lateral mesocoxal foveae (Fig. 3C); length of abdominal tergite and visible sternite VI approximately twice as long as V (Fig. 1); species only known from Western Australia (Fig. 5).

#### Description.

**Figure 2.**
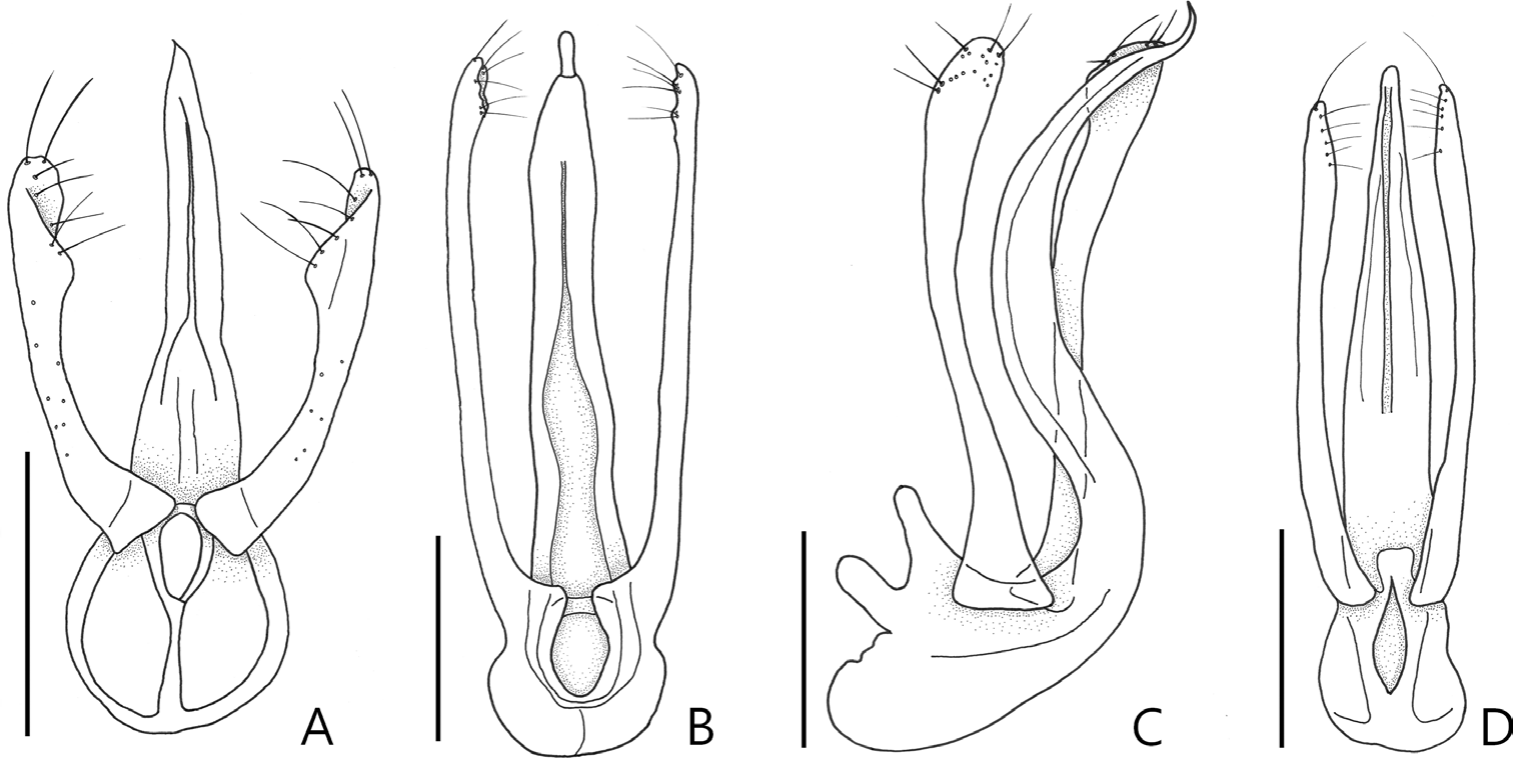
Male genitalia of the species of *Porongurup* gen. nov. **A***P. angulatus* sp. nov., ventral view **B***P. clarkei* sp. nov., ventral view **C***P. clarkei* sp. nov., lateral view **D***P. tenuis* sp. nov., ventral view. Scale bars: 0.1 mm.

**Figure 3.**
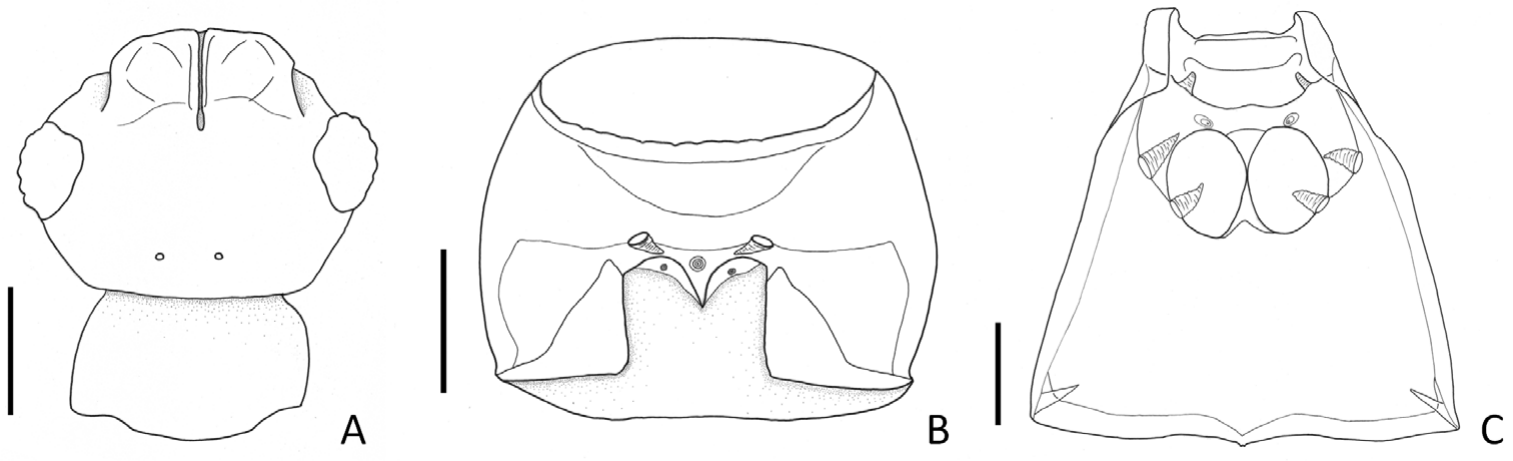
Generic characters of *Porongurup angulatus* sp. nov. **A** Head with frontal sulcus, dorsal view **B** Prosternum, ventral view **C** meso- metaventrite, ventral view. Scale bar: 0.1 mm.

**Figure 4.**
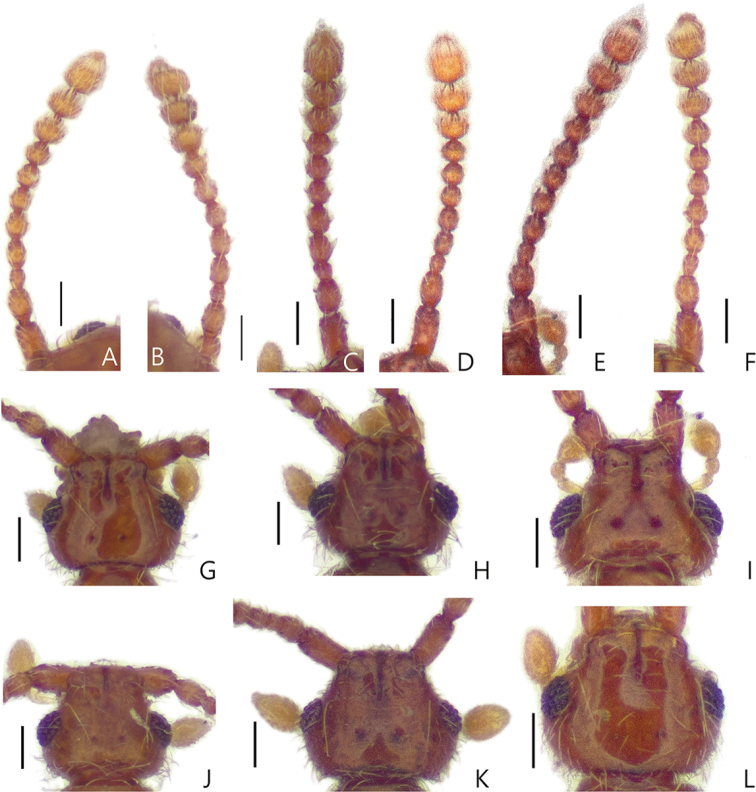
Antennae of *Porongurup angulatus* sp. nov. **A** male **B** female. Antennae of *P. clarkei* sp. nov. **C** male **D** female. Antennae of *P. tenuis* sp. nov. **E** male **F** female. Male head dorsal view **G***P. angulatus* sp. nov. **H***P. clarkei* sp. nov. **I***P. tenuis*. sp. nov. Female head dorsal view **J***P. angulatus* sp. nov. **K***P. clarkei* sp. nov. **L***P. tenuis* sp. nov. Scale bar: 0.1 mm. *The distinctness and position of the vertexal foveae may be affected by the angle of view and condition of the specimens.

Small body size, 1.6–2.0 mm. Body yellowish to reddish brown. *Head*. Head with distinct narrow frontal sulcus, and distinct vertexal foveae (Fig. 4G–L). Male head triangular, broader than long, widest across eyes. Female head more rounded than that of male (Fig. 4G–L). *Thorax.* Prosternum broader than long, widest at midpoint of prosternum (Fig. 3B). *Abdomen.* Length of abdominal segment VI approximately two times longer than V (Fig. 1). *Aedeagus.* Median lobe of male genitalia slender, apically sharp and narrow. Phallobase rounded. Parameres symmetrical and slender with more than five setae apically (Fig. 2).

#### Etymology.

*Porongurup* gen. nov. is named for Porongurup National Park, where one of the species was collected. Gender: Masculine.

#### Distribution.

Western Australia.

#### Comments about secondary sexual characters.

Male specimens have larger eyes than females (Fig. 4G–L). The males of *Porongurup clarkei* sp. nov. and *P. tenuis* sp. nov. have longer elytra and fully developed hind wings, but female specimens have the elytra almost half the size of those of the males, and the hind wings are reduced (Fig. 1). *Porongurup angulatus* sp. nov. has fully developed hind wings in both sexes (Fig. 1A, D).

#### Comments about related taxa.

Based on the thoracic foveal system and the longer abdominal segment VI, *Porongurup* gen. nov. is closest to the genus *Nornalup* Park & Chandler, 2017. However, all specimens of *Porongurup* gen. nov. do not have a median metasternal foveae (Fig. 3C) and have a narrow frontal sulcus (Fig. 3A). The aedeagal form is also different from that of *Nornalup* in the elongate triangular shape of both the median lobe and the parameres (Fig. 2). The parameres of *Nornalup* species are as wide as the median lobe, and the median lobe is parallel-sided in dorsal view (Park and Chandler 2017: fig. 4d–i).

##### Key to species of the genus *Porongurup* gen. nov.

**Table:** 

1	Antennomere III subquadrate, as long as wide (Fig. 4A, B); female elytra longer than wide with fully developed hind-wings (Fig. 1A, D); length of abdominal segment VI 2.0–2.5 times as long as V (Fig. 1A, D)	***Porongurup angulatus* sp. nov.**
–	Antennomere III rectangular, longer than wide (Fig. 4C–F); female elytra as long as wide with reduced hind-wings (Fig. 1E, F); length of abdominal segment VI approximately three times as long as V (Fig. 1B, C, E, F)	**2**
2	Median lobe of male genitalia with small articulated extension apically (Fig. 2B: arrowed)	***P. clarkei* sp. nov.**
–	articulated extension of median lobe of male genitalia absent (Fig. 2D: arrowed)	***P. tenuis* sp. nov.**

### Porongurup angulatus sp. nov.

Taxon classificationAnimaliaColeopteraStaphylinidae

5B1FFBDD-67B8-5D99-BA28-AA9B20C7CD5C

http://zoobank.org/69F25550-F02F-4F92-8662-201AAF05FFDC

**Figure 5.**
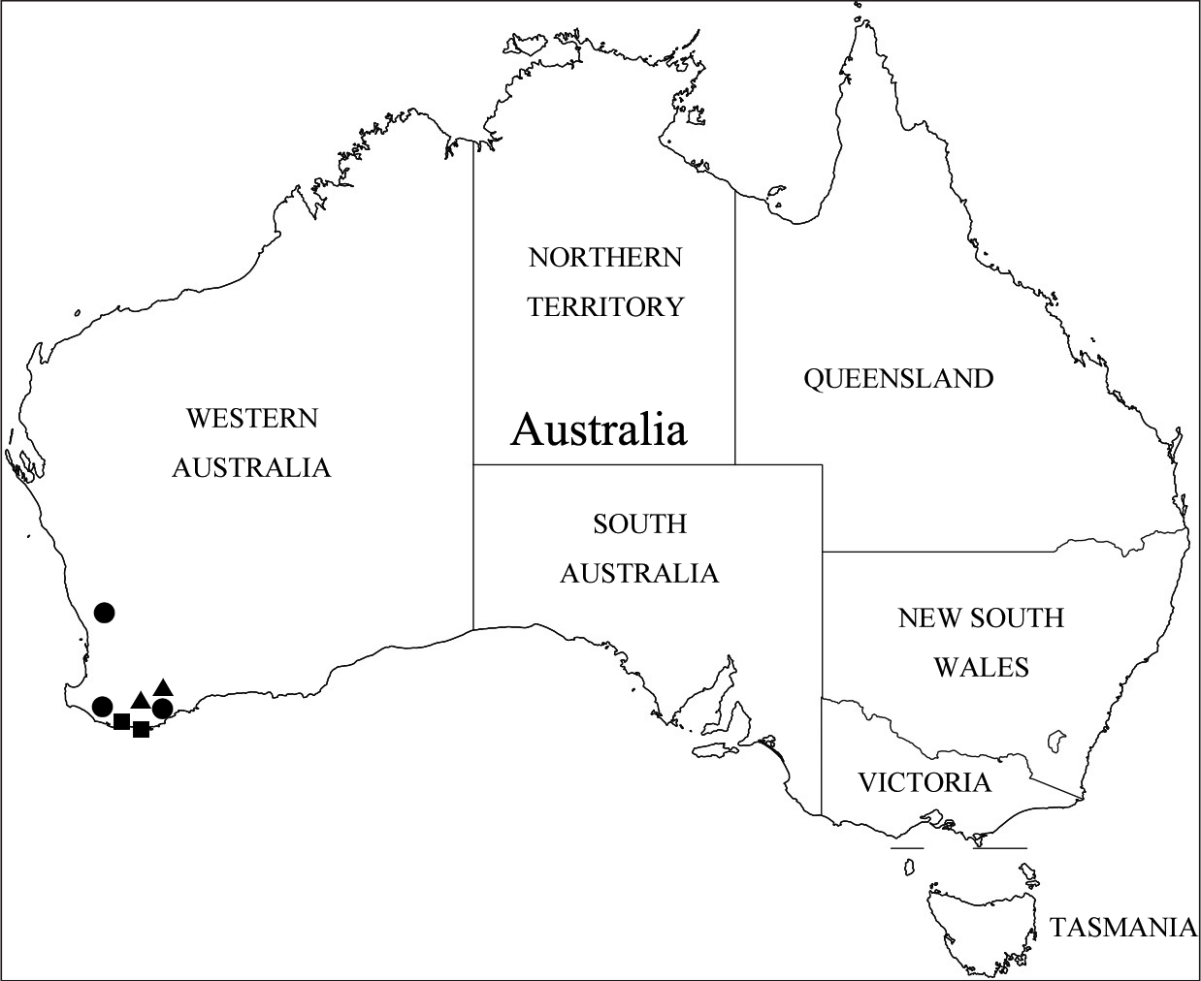
Collection localities of *Porongurup* gen. nov. Key: *P. angulatus* sp. nov., circles; *P. clarkei* sp. nov., triangle; *P. tenuis* sp. nov., squares.

Figs 1A , 1D , 2A , 3 , 4A–B , 4G , 4J , 5

#### Type material.

***Holotype*. Australia: Western Australia (WA)**: 1♂, aedeagus dissected and mounted in euparal on clear plastic card, “**AUSTRALIA: Western Australia**: Porongurup N.P., Wansborough Walk at The Pass, 450 m, 34°40.69'S, 117°51.245'E, 6 VIII 2004, karri forest (*Eucalyptus diversicolor*), mostly young-growth; FMHD#2004-147, Berlese, leaf & log litter, A. Newton & M. Thayer 1116”. ***Paratypes* (*N* = 12; 6 males, 6 females). Australia: Western Australia**: 1♀ (FMNH, slide mounted), 40 km ESE Manjimup, Cup Road, 6 VII 1980, FMHD# 80-400, karri bark litter, S. & J. Peck; 1♂1♀ (UNHC, 1♀ slide mounted), 1♂ aedeagus dissected and mounted in Euparal on clear plastic card, Windy Harbour, 27 km S Northcliffe, 8 VII 1980, coastal scrub litter, S. & J. Peck; 1♂2♀ (FMNH, 1♀ slide mounted), Avon Valley N.P., 1.3 km from entrance, 420 m, 31°38.79'S, 116°17.94'E, 27 VII–13 VIII 2004, marri-jarrah (*Eucalyptus calophylla-E. marginata*) woodland; FMHD#2004-103, flight intercept trap, A. Newton & M. Thayer, 1102; 1♂ (CBNUIC, slide mounted), Walpole-Nornalup N.P., Giant Tingle Tree area, 190 m, 34°58.88'S, 116°47.42'E, 2 VIII 2004, tingle-*Allocasuarina*-karri (*Eucalyptus diversicolor*) forest; FMHD#2004-132, Berlese, leaf & log litter, Newton, Thayer, Clarke 1110; 1♂1♀ (CBNUIC), 1♂ aedeagus dissected and mounted in Euparal on clear plastic card, 54 km SE Manjimup, 22 VI/26 VI 1980, S. &J. Peck. jarrah forest litter; 1♂ (FMNH), aedeagus dissected and mounted in Euparal on clear plastic card, Pemberton, Brockman N.P., 19 VII 1980, FMHD#80-406, karri base & fungi litter, S. & J. Peck; 1♂ (FMNH), aedeagus dissected and mounted in Euparal on clear plastic card, Warren N.P., Bicentennial Tree vic., 120 m, 34°29.73'S, 115°58.62'E, 30 VII–10 VIII 2004, karri forest (*Eucalyptus diversicolor*); FMHD#2004-114, flight intercept trap, Newton, Solodovnikov, Thayer 1105; 1♀ (UNHC) Avon Valley N.P., Governor’s Drive, 1.2 km from Forty-one Mile Rd., 260 m, 31°36.57'S, 116°15.04'E, 27 VII 2004, *Eucalyptus wandoo* woodland; FMHD#2004-102, Berlese, leaf & log litter, A. Solodovnikov, D. Clarke et al. 1101.

#### Diagnosis.

This species can be distinguished from *Porongurup clarkei* sp. nov. by the smaller size of the male genitalia (Fig. 2), the subquadrate antennomere III (Fig. 4A, B), and the length of abdominal segment VI is approximately twice as long as V. This species is also separated from *Porongurup tenuis* sp. nov. by the length of abdominal segment VI being twice as long as V (Fig. 1), and the parameres of the male genitalia approximately are twice as wide as those of the other species (Fig. 2).

#### Description.

Length 1.7–1.9 mm (Fig. 1A, D). *Head.* Head in dorsal view with deep frontal sulcus and vertexal foveae (Fig. 4G). Antennomeres II longer than wide, III subquadrate, smallest of the antennomeres, IV and V longer than wide, VI and VIII as long as wide, IX and X transverse (Fig. 4A, B). *Abdomen*. Length of abdominal segment VI twice as long as V in both sexes (Fig. 1A, D). *Aedeagus.* Median lobe of male genitalia elongated triangular, lacking articulated extension at tip of apex. Phallobase rounded in dorsal view. Parameres as wide as at middle of median lobe (Fig. 2A).

#### Etymology.

This species name refers to the sub-apically angulate parameres of the male genitalia.

#### Distribution.

Western Australia (Fig. 5, circles).

#### Habitat.

Specimens of this species were collected using flight intercept traps, or were taken by sifting leaf, bark, log, or fungus in *Eucalyptus* forests.

### Porongurup clarkei sp. nov.

Taxon classificationAnimaliaColeopteraStaphylinidae

931DA0B4-0EBD-5F03-B5D8-F602DCE57BAE

http://zoobank.org/11556FE3-6FDF-4265-999C-C160B8F6F17E

Figs 1B , 1E , 2B–C , 4C–D , 4H , 4K , 5

#### Type material.

***Holotype*. Australia: Western Australia (WA)**: 1♂, aedeagus dissected and mounted in Euparal on clear plastic card, “**AUSTRALIA: Western Australia**: Stirling Range N.P., Toolbrunup Peak Track, 480–520 m, 34°23.4'S, 118°03.3'E, 5 VIII 2004, *Eucalyptus* forest & mallee; FMHD#2004-146, Berlese, leaf & log litter, Clarke & Grimbacher 1115”. ***Paratypes* (*N* = 11; 7 males, 4 females). Australia: Western Australia**: 1♂2♀ (FMNH, 1♀ slide mounted), Stirling Range N.P., Toolbrunup Peak Track, 430–485m, 34°23.5'S, 118°03.65'E, 5 VIII 2004, mallee *Eucalyptus*; FMHD#2004-145, Berlese, water-washed soil, 0–18 cm, D. Clarke 1114; 5♂ (UNHC), 1♂ aedeagus dissected and mounted in Euparal on clear plastic card, Porongorup N.P., Bolganup Creek, 12 VI 1980, Berlese, bark & fungi karri tree bases, S. &J. Peck; 1♂ (CBNUIC, aedeagus dissected and mounted in Euparal on clear plastic card, Stirling Range N.P., Toolbrunup Tr. WA, 10 VI 1980, Berlese, rotted logs & moss, S. & J. Peck, SBP47; 1♀(FMNH), Stirling Range N.P., Toolbrunup Tr. WA, 10 VI 1980, Berlese, fungi on Euc. Trunks, S. & J. Peck, SBP45; 1♀(UNHC), Porongorup N.P., Bolganup Creek, 12 VI 1980, Berlese, bark & fungi on karri tree bases, S. & J. Peck, SBP53.

#### Diagnosis.

This species can be distinguished from *Porongurup angulatus* sp. nov. by antennomere 3 being longer than wide (Fig. 4C), and abdominal segment VI being approximately three times longer than V (Fig. 1B, E). This species is also separated from *Porongurup tenuis* sp. nov. by the median lobe of the male genitalia having a small digit at the apex (Fig. 2B: arrowed).

#### Description.

Length 1.6–1.9 mm (Fig. 1B, E). *Head.* Head in dorsal view with both shallow frontal sulcus and vertexal foveae. Male and female antennae are almost identical in length (Fig. 4C, D). Antennomeres II longer than wide, III subquadrate and smallest, IV—VIII longer than wide, IX and X transverse (Fig. 4C, D). *Elytra*. Male elytra longer than wide, female elytra shorter, as long as wide (Fig. 1B, E). *Abdomen*. Abdominal segment VI approximately three times longer than V (Fig. 1B, E). *Aedeagus.* Median lobe of male genitalia as long as parameres with apical articulated extension. Phallobase rounded in dorsal view. Parameres symmetrical (Fig. 2B, C).

#### Etymology.

This species is named for one of the collectors of the holotype, the staphylinid specialist Dave J. Clarke.

#### Distribution.

Western Australia (Fig. 5, triangles).

#### Habitat.

Specimens of this species were collected by sifting leaf, log, bark, moss or fungus litter in *Eucalyptus* forests, or were taken by Berlese funnel water-washed soil.

### Porongurup tenuis sp. nov.

Taxon classificationAnimaliaColeopteraStaphylinidae

7AD22A25-0157-5449-B47E-A186E4301527

http://zoobank.org/04ECBDD6-4E5C-4450-BD21-12856B1D1334

Figs 1C , 1F , 2D , 4E-F , 4I , 4L , 5

#### Type material.

***Holotype*. Australia: Western Australia (WA)**: 1♂, aedeagus dissected and mounted in Euparal on clear plastic card, “**AUSTRALIA: Western Australia**: Walpole-Nornalup N.P., 1.4 km NE Mandalay Beach, 20 m, 34°59.76'S, 116°32.94'E, 3–9 VIII 2004, mixed forest on old dunes; FMHD#2004-140, flight intercept trap, A. Newton, M. Thayer, A. Solodovnikov 1112”. ***Paratypes* (*N* = 5; 1 male, 4 females). Australia: Western Australia**: 1♂(FMNH), elytra and aedeagus dissected in micro vial, Mt. Clare, 12 km, W Walpole, 20 XII 1976, JKethley, FM#76-517, Ber. #151, Euc. cornuta litter; 1♀(FMNH), Walpole-Nornalup N.P., Anderson Rd. near Valley of the Giants Rd., 120 m, 34°59.48'S, 116°52.35'E, 2 VIII 2004, tingle-*Allocasuarina*-karri (*Eucalyptus diversicolor*) forest; FMHD#2004-137, Berlese, leaf & log litter, A. Newton, M. Thayer, et al. 1111; 1♀ (CBNUIC, slide mounted), Walpole-Nornalup N.P., Giant Tingle Tree area, 190 m, 34°58.88'S, 116°47.42'E, 2–9 VIII 2004, tingle-*Allocasuarina*-karri (*Eucalyptus diversicolor*) forest; FMHD#2004-130, flight intercept trap, Newton, Solodovnikov, Thayer, 1110; 2♀ (1, UNHC; 1 FMNH), Walpole N.P. Hilltop Rd., 21 VI 1980, Berlese, karri & tingle tree litter, S. & J. Peck.

#### Diagnosis.

This species can be distinguished from *Porongurup angulatus* sp. nov. by the elytra being as long as wide (Fig. 1C, F). It also differs from *P. clarkei* sp. nov. by lacking the articulated extension at the apex of the median lobe of the male genitalia (Fig. 2D).

#### Description.

Length 1.8–2.0 mm (Fig. 1C, F). *Head.* Head in dorsal view with both deep and narrow frontal sulcus and vertexal foveae. Male antennae longer than those of female (Fig. 4E, F). Antennomeres II longer than wide, III subquadrate and smallest, IV and V longer than wide, VI—VIII as long as wide, IX and X transverse (Fig. 4E, F). *Elytra*. Male elytra longer than wide, female elytra shorter than those of male (Fig. 1C, F). *Abdomen*. Male abdominal segment VI approximately three times as long as V, female with segment VI 1.5 times longer than V (Fig. 1C, F). *Aedeagus.*Median lobe of male genitalia as long as parameres, apex sharp and narrow. Phallobase oval in dorsal view. Parameres symmetrical (Fig. 2D).

#### Etymology.

This species name refers to the elongate slender parameres of the male genitalia.

#### Distribution.

Western Australia (Fig. 5, squares).

#### Habitat.

Specimens of this species were collected using flight intercept traps, or by sifting leaf or log litter.

## Supplementary Material

XML Treatment for Porongurup

XML Treatment for Porongurup angulatus

XML Treatment for Porongurup clarkei

XML Treatment for Porongurup tenuis
